# Supplementary material for: Locomotion-induced ocular motor behavior in larval Xenopus is developmentally tuned by visuo-vestibular reflexes
Source: Nat Commun. 2022 May 26;13:2957. doi: 10.1038/s41467-022-30636-6 (PMC9135768; doi:10.1038/s41467-022-30636-6)
Supplement: Supplementary file 1 — Supplementary Information [file 41467_2022_30636_MOESM1_ESM.pdf]

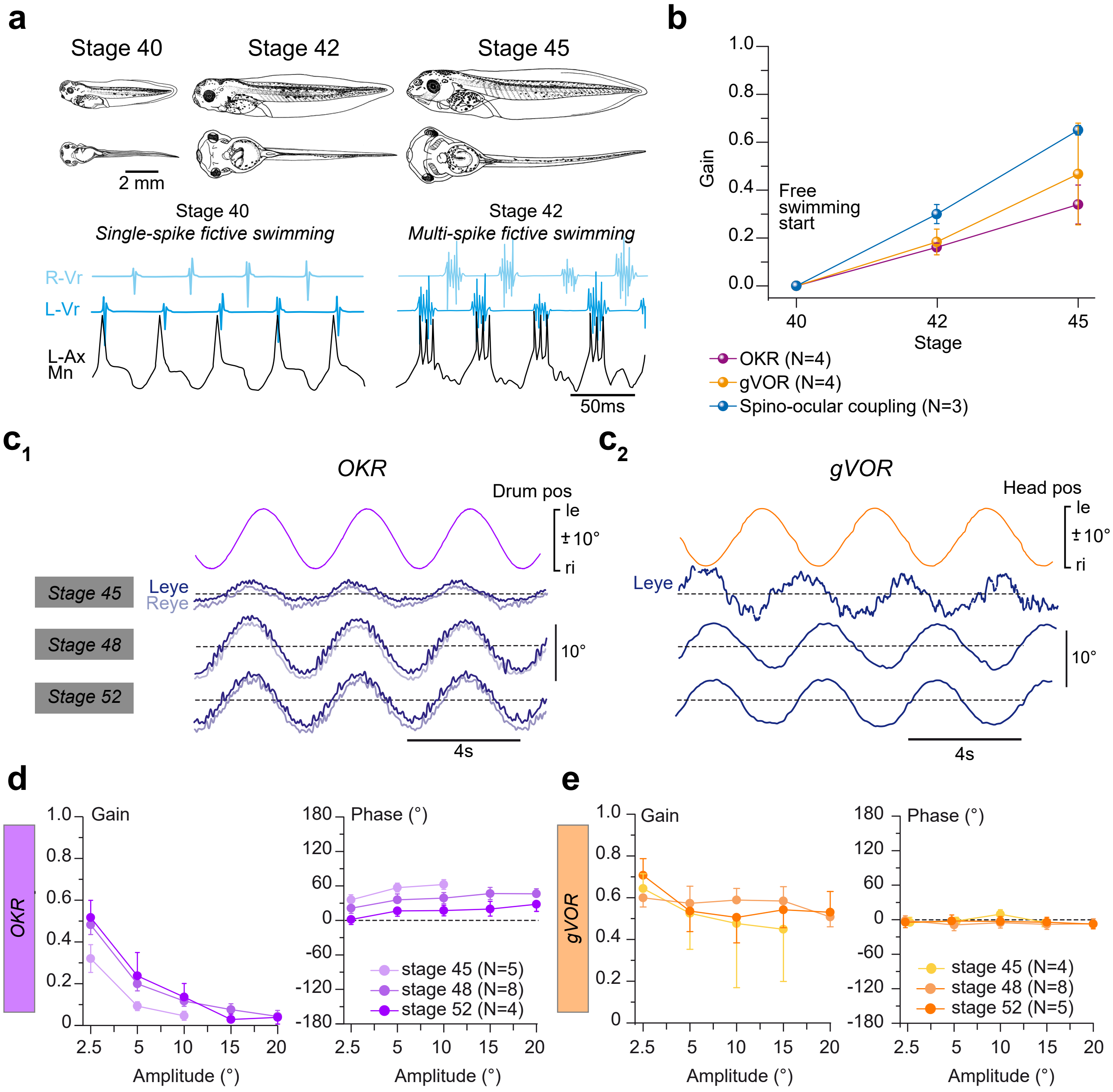

Supplementary figure 1

Supplementary Figure 1: Ontogenetic improvement of gaze-stabilizing ocular motor behaviors. (a) Developmental switch from single-spike to multi-spike burst activity in spinal axial motoneurons (Ax Mn) between stage 40 and 42 (modified from [29]). **(b)** Eye position (pos) gain for the OKR (N= 4 at stage 40, 42, 45), gVOR (N= 4 at stage 40, 42, 45) and spino-ocular (N=3 at each stages) motor coupling at stage 40, 42 and 45 (mean ± SEM). **(c)** Movements of the left (Leye, dark blue) and right eye (Reye, light blue) typical for the optokinetic (OKR, $\mathbf{c_1}$) and gravitoinertial vestibulo-ocular reflex (gVOR, $\mathbf{c_2}$) at stage 45, stage 48 and stage 52; ocular motor responses were elicited by sinusoidal rotation of black/white vertical stripes or downward-upward head roll motion at 0.25 Hz and positional excursions (pos) of ± 10°. **(d)** Eye position gain (left) and phase (right) for the OKR as function of stimulus amplitude (mean ± SEM) at stages 45 (N = 5), 48 (N = 8) and 52 (N = 4). **(e)** Eye position gain (left) and phase (right) for the gVOR as function of stimulus amplitude (mean ± SEM) at stages 45 (N = 4), 48 (N = 8) and 52 (N = 5). le, ri, leftward, rightward movement; R, L, right, left. Source data are provided as a Source Data file.

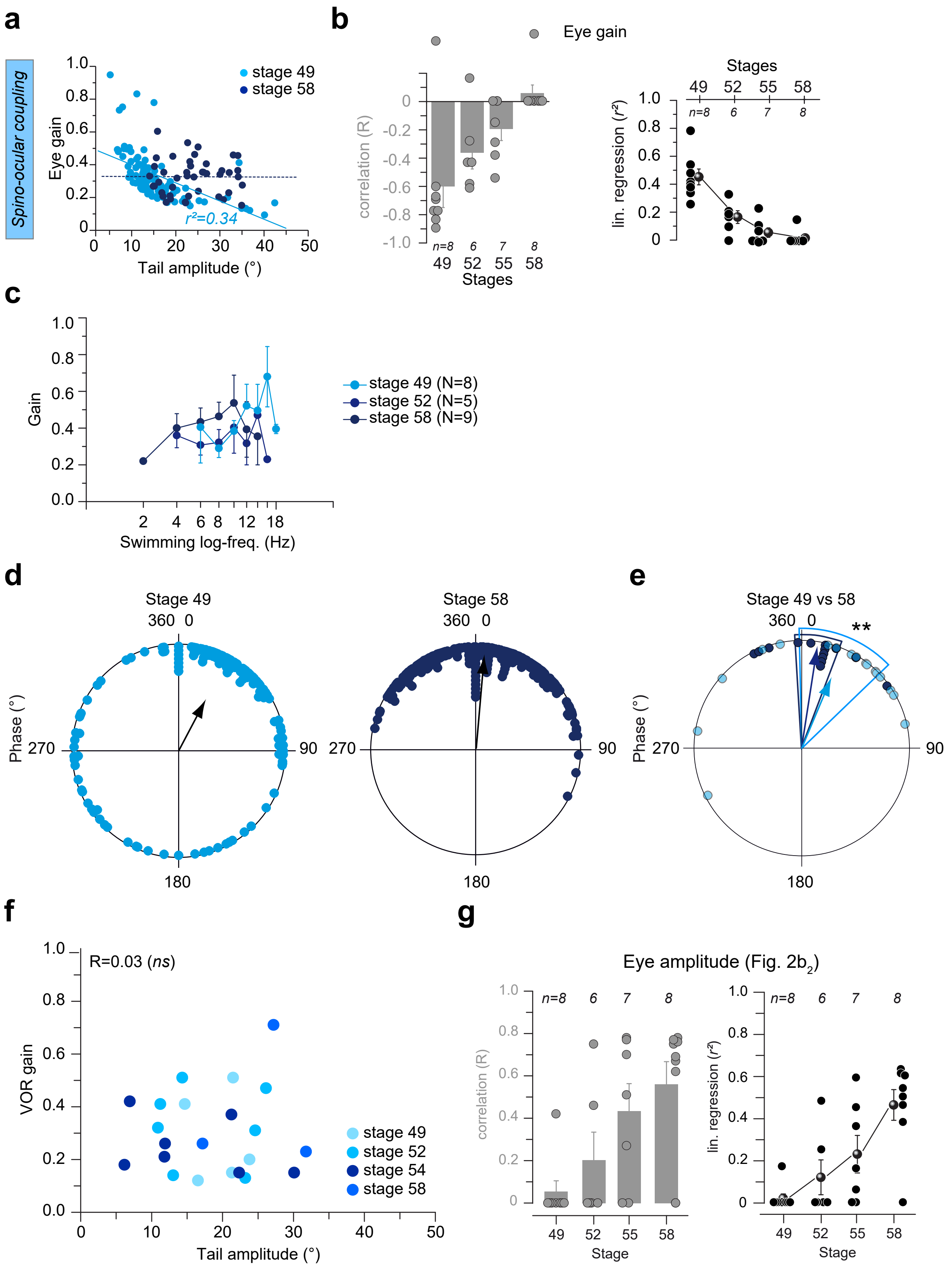

Supplementary figure 2

Supplementary Figure 2: Progressive developmental enhancement of spino-ocular motor coupling. (a) Scatterplot representing eye position gain as function of tail undulation amplitude at stage 49 and 58. **(b)** Histogram (grey bars) of correlation coefficients (*R*) and linear (lin.) regression (r^2, black circles) for eye position gains at stage 49 (N = 8), 52 (N = 6), 55 (N = 7) and 58 (N = 8). Data are presented as mean values ± SEM. **(c)** Bode analysis of the eye position gain as function of the tail undulation frequency at stage 49 (N = 8), 52 (N = 5), 58 (N = 9) (mean ± SEM). **(d)** Polar plots of phase relations between eye and tail positions for representative examples at stage 49 (left, light blue) and stage 58 (right, dark blue). **(e)** Polar plot of average phase relations (mean ± 95% confidence intervals; simplified plot shown in inset on the right of Fig. $2b_3$) at stage 49 (n = 17 swimming episodes from N = 11 animals) and stage 58 (n = 14 swimming episodes from N = 12 animals). Non-uniformity distribution has been tested with Moore's modified Rayleigh test with $p < 0.001$ for stage 49 and 58. Significance of the difference between mean values of both stages has been tested by Hotelling's two sample statistical test with $p = 0.01$. le, ri, leftward, rightward movement. (**f**) Scatter plot of the aVOR gain with respect to swim-related tail undulation amplitudes at stage 45, 49, 52 and 58. Two-tailed *p*-values were calculated to estimate the Pearson coefficient correlation (R) significance, with $p = 0.89$, indicating a non-significant result (ns). (**g**) Histogram (grey bars) of correlation coefficients (*R*) and linear (lin.) regression (r^2, black circles) for eye amplitude gains at stage 49 (N = 8), 52 (N = 6), 55 (N = 7) and 58 (N = 8). Data are presented as mean values ± SEM. Source data are provided as a Source Data file.

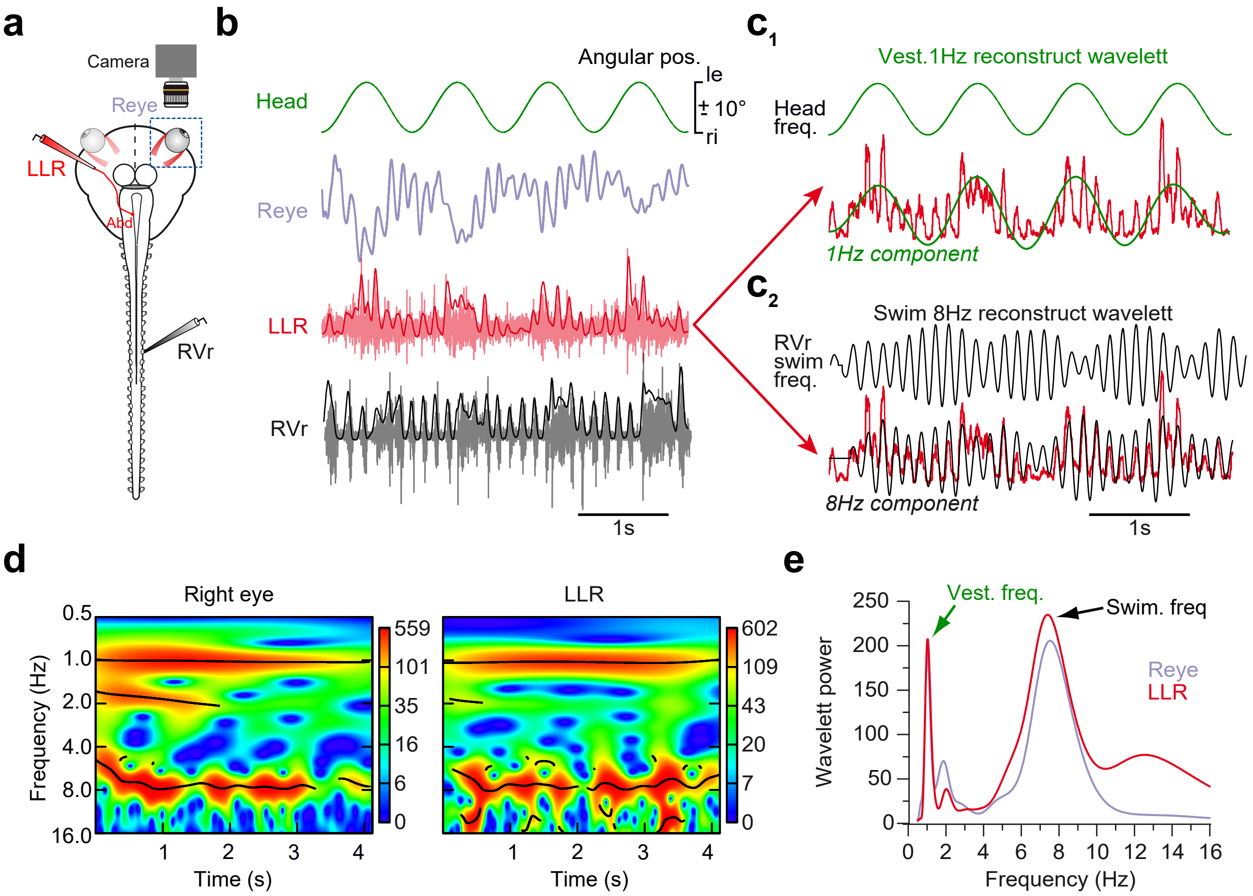

Supplementary Figure 3

Supplementary Figure 3: Eye movements and *lateral rectus* motor nerve discharge during vestibular stimulation and concurrent fictive swimming. (a) Schematic depicting the simultaneous recording of ocular movements of the right eye (Reye), spike discharge of the left *lateral rectus* (LLR, red) nerve and a right spinal ventral root (RVr, black) at stage 52. (**b**) Representative recordings of eye movements, LLR and RVr discharge during fictive swimming and horizontal head rotation (1 Hz, ± 10°) as spike activity (light red and grey) and after signal integration (dark red, black; see methods). **(c)** Wavelet reconstruction of vestibular stimulus- (**c_1**) and fictive swim-related (**c_2**) frequency components, extracted from the integrated LLR spike discharge after spectral analysis; the 1 Hz component (green trace superimposed on the red LLR discharge integral) matches the head rotation waveform (top panel), whereas the 8 Hz frequency component (black trace superimposed on the red LLR discharge integral) coincides with the burst frequency of the fictive swim-related RVr spike discharge (bottom panel). **(d, e)** Spectrograms (**d**) and periodogram (**e**) depicting the vestibular motion stimulus-related response at 1 Hz (Vest. freq.) and fictive swim-related response at a frequency of 8 Hz (Swim. freq.), expressed as movement of the right eye (light violet) and spike discharge of the LLR nerve (red) obtained from **b** and **c**. Abd, abducens motor nucleus; le, ri, leftward, rightward movement.

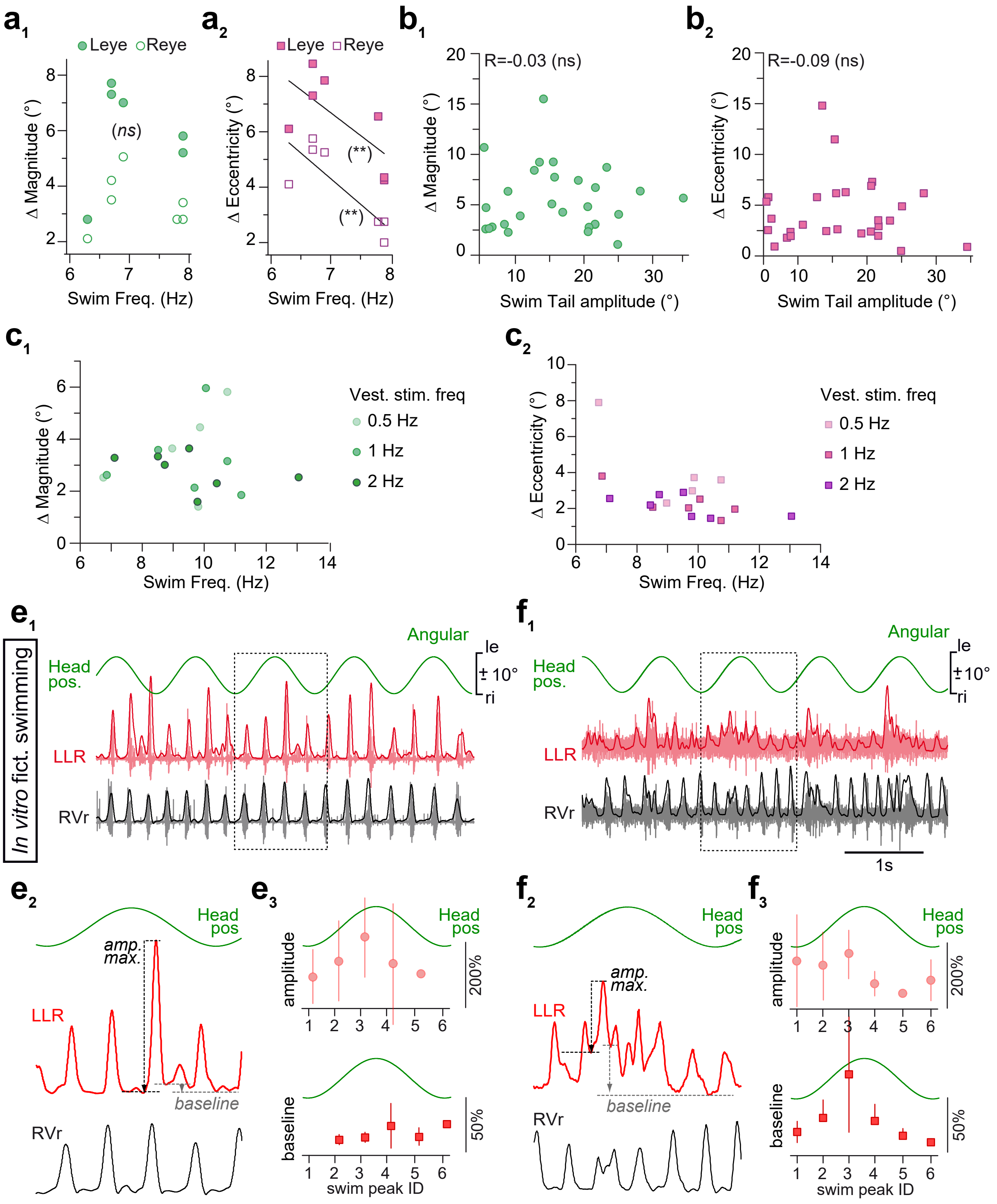

Supplementary Figure 4

Supplementary Figure 4: Impact of swim parameters on concurrent eye motion performance. (**a**) Scatter plots of Δ eye motion magnitude (**a_1**) and Δ eye position eccentricity (**a_2**) as function of the swim frequency for the left and right eye (Leye, Reye) obtained from the example shown in Fig. 5a (n = 7 cycles). Pearson coefficient correlations were rendered non-significant for Δ eye motion magnitude following a two-tailed test: p = 0.56 (Leye) and p = 0.76 (Reye). Pearson coefficient correlations were non-significant for Δ eye position eccentricity of the Leye p= 0.09 and significant for the Reye p = 0.02 (**b**) Scatter plots of Δ eye motion magnitude (**b_1**) and Δ eye position eccentricity (**b_2**) as function of the swim-related tail undulation amplitude. Pearson coefficient correlations were non-significant for Δ eye motion magnitude and Δ eye position eccentricity following two-tailed tests: p = 0.87 and p = 0.64 respectively (**c**) Scatter plot of Δ eye motion magnitude (**c_1**) and Δ eye position eccentricity (**c_2**) as function of the swim frequency for different vestibular stimulus frequencies at 0.5 Hz (n = 5 swimming episodes from N = 4 animals), 1 Hz (n = 6 swimming episodes from N = 4 animals) and 2Hz (n = 7 swimming episodes from N = 3 animals). (**e, f**) Representative episode (**e_1, f_1**) and vestibular motion cycle-related examples (**e_2, f_2**) of the spike discharge recorded from the left lateral rectus (LLR) nerve during horizontal head rotation (1 Hz, ± 10°) and concurrent fictive swimming, visible as rhythmic bursting of the right spinal ventral root (RVr), and after signal integration (dark red, black in **e_1** and **f_1**). Comparable to the eye motion modulation shown in Fig. 5, sinusoidal head rotation at 1 Hz modulated the swim-related burst amplitude in the LLR nerve discharge (**e_3, f_3**; light red dots in top panels) either separately or conjointly with the LLR baseline discharge (**e_3, f_3**; dark red squares in bottom panels). Data are presented as mean values ± SEM. Source data are provided as a Source Data file.

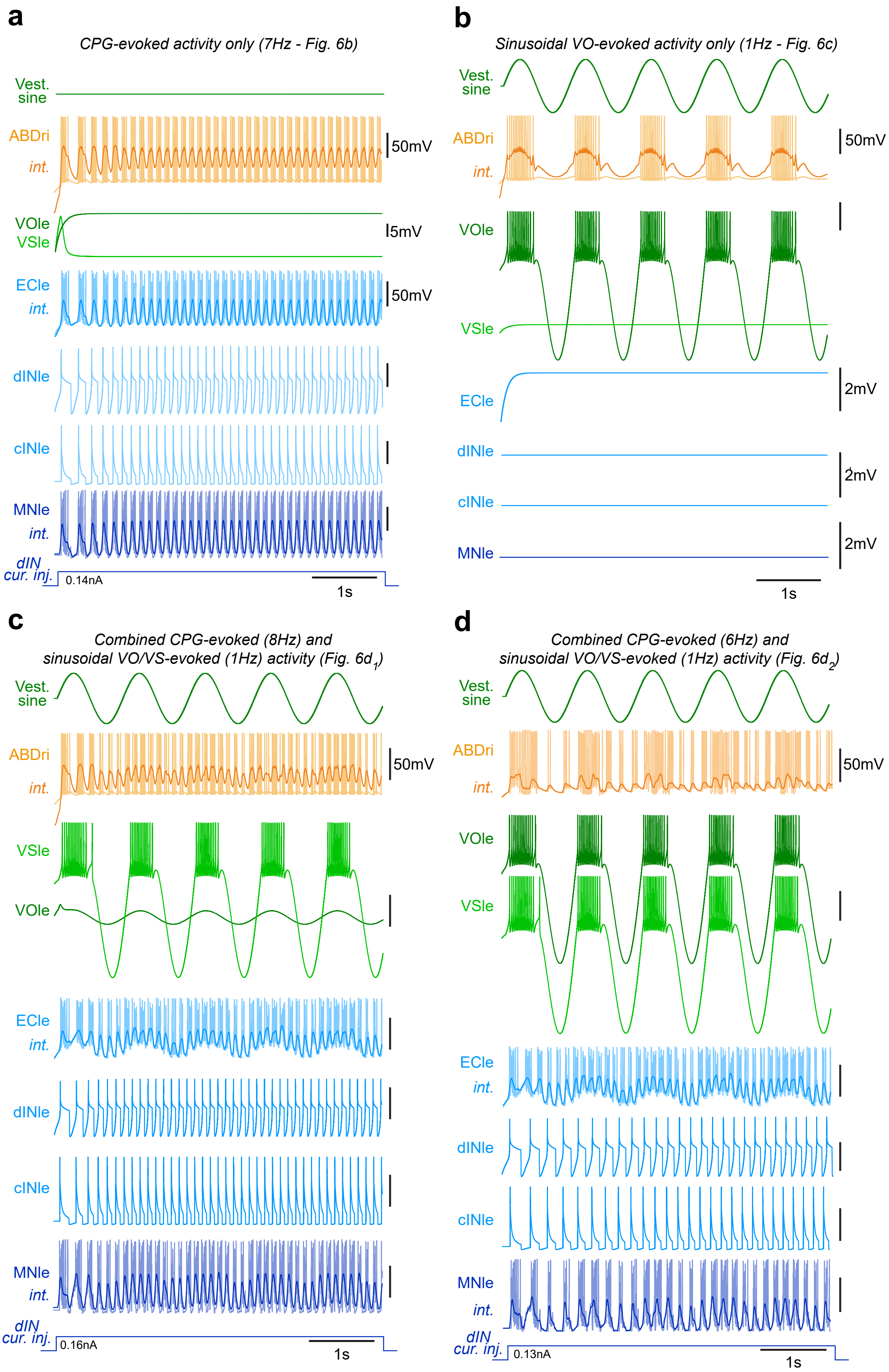

Supplementary Figure 5

Supplementary Figure 5: Additional neuronal model traces for visuo-vestibulo-ocular motor computations. (**a-d**) Discharge patterns from all model neurons of a hemi-circuit, highlighted in the scheme of Fig. 6a, in response to a bilateral rhythmic current injection of 0.15 nA into dIN (**a**, corresponding to Fig. 6b), in response to sinusoidal current injection at 1 Hz into the VO neuron only (**b**, corresponding to the Fig. 6c), in response to sinusoidal current injection into the VO and VS neuron, combined with current injection > 0.16 nA into dIN (**c**, corresponding to Fig. $6d_1$), in response to sinusoidal current injection into the VO and VS neuron, combined with current injection < 0.16 nA into dIN (**d**, corresponding to Fig. $6d_2$). le, left; ri, right; int., integrated trace. Scale bars represent 50 mV unless stated otherwise.
