# Supplementary material for: Locomotion-induced ocular motor behavior in larval Xenopus is developmentally tuned by visuo-vestibular reflexes
Source: Nat Commun. 2022 May 26;13:2957. doi: 10.1038/s41467-022-30636-6 (PMC9135768; doi:10.1038/s41467-022-30636-6)
Supplement: Supplementary file 3 — Reporting Summary [file 41467_2022_30636_MOESM3_ESM.pdf]

nature research

Corresponding author(s): Dr. François M. Lambert

Last updated by author(s): Apr 14, 2022

Reporting Summary

Nature Research wishes to improve the reproducibility of the work that we publish. This form provides structure for consistency and transparency in reporting. For further information on Nature Research policies, see our Editorial Policies and the Editorial Policy Checklist.

Statistics

For all statistical analyses, confirm that the following items are present in the figure legend, table legend, main text, or Methods section.

n/a	Confirmed	
☐	☒	The exact sample size (*n*) for each experimental group/condition, given as a discrete number and unit of measurement
☐	☒	A statement on whether measurements were taken from distinct samples or whether the same sample was measured repeatedly
☐	☒	The statistical test(s) used AND whether they are one- or two-sided<br>*Only common tests should be described solely by name; describe more complex techniques in the Methods section.*
☒	☐	A description of all covariates tested
☒	☐	A description of any assumptions or corrections, such as tests of normality and adjustment for multiple comparisons
☐	☒	A full description of the statistical parameters including central tendency (e.g. means) or other basic estimates (e.g. regression coefficient) AND variation (e.g. standard deviation) or associated estimates of uncertainty (e.g. confidence intervals)
☐	☒	For null hypothesis testing, the test statistic (e.g. F, t, r) with confidence intervals, effect sizes, degrees of freedom and P value noted<br>*Give P values as exact values whenever suitable.*
☒	☐	For Bayesian analysis, information on the choice of priors and Markov chain Monte Carlo settings
☒	☐	For hierarchical and complex designs, identification of the appropriate level for tests and full reporting of outcomes
☒	☐	Estimates of effect sizes (e.g. Cohen's d, Pearson's r), indicating how they were calculated

Our web collection on statistics for biologists contains articles on many of the points above.

Software and code

Policy information about availability of computer code

Data collection	Tracking of the eyes and tail was automated using a custom-made software, programmed in Python 3.5 environment using opencv library. PyQtgraph was used for graphical user interface. Electrophysiological signals was vizualised and stored on a computer by a commercial software Spike2 v7 (Cambridge Electronic Design, UK).
Data analysis	Raw data of video or electrophysiological recordings were processed off-line and analyzed with Dataview v11.5.1 (by W.J. Heitler, University of St Andrews, Scotland). A custom-built R script from “wavelettComp” package (Computational Wavelet analysis; R package v1.0; http://CRAN.R-project.org/package=WavelettComp) was used to perfom spectral analysis

For manuscripts utilizing custom algorithms or software that are central to the research but not yet described in published literature, software must be made available to editors and reviewers. We strongly encourage code deposition in a community repository (e.g. GitHub). See the Nature Research guidelines for submitting code & software for further information.

Data

Policy information about availability of data

All manuscripts must include a data availability statement. This statement should provide the following information, where applicable:

- Accession codes, unique identifiers, or web links for publicly available datasets
- A list of figures that have associated raw data
- A description of any restrictions on data availability

The datasets generated and analyzed during the current study are available at http://dx.doi.org/10.17632/pgmb54b89b.3

Field-specific reporting

Please select the one below that is the best fit for your research. If you are not sure, read the appropriate sections before making your selection.

☒ Life sciences ☐ Behavioural & social sciences ☐ Ecological, evolutionary & environmental sciences

For a reference copy of the document with all sections, see nature.com/documents/nr-reporting-summary-flat.pdf

Life sciences study design

All studies must disclose on these points even when the disclosure is negative.

Sample size	Significance of statistical tests was used to determine the best sample size;
Data exclusions	Some datas from oculomotor or motor behavior were excluded if the tracking of eye and tail movement was not enough relevant.
Replication	*Describe the measures taken to verify the reproducibility of the experimental findings. If all attempts at replication were successful, confirm this OR if there are any findings that were not replicated or cannot be reproduced, note this and describe why.*
Randomization	*Describe how samples/organisms/participants were allocated into experimental groups. If allocation was not random, describe how covariates were controlled OR if this is not relevant to your study, explain why.*
Blinding	*Describe whether the investigators were blinded to group allocation during data collection and/or analysis. If blinding was not possible, describe why OR explain why blinding was not relevant to your study.*

Reporting for specific materials, systems and methods

We require information from authors about some types of materials, experimental systems and methods used in many studies. Here, indicate whether each material, system or method listed is relevant to your study. If you are not sure if a list item applies to your research, read the appropriate section before selecting a response.

Materials & experimental systems

n/a	Involved in the study	
☒	☐	Antibodies
☒	☐	Eukaryotic cell lines
☒	☐	Palaeontology and archaeology
☐	☒	Animals and other organisms
☒	☐	Human research participants
☒	☐	Clinical data
☒	☐	Dual use research of concern

Methods

n/a	Involved in the study	
☒	☐	ChIP-seq
☒	☐	Flow cytometry
☒	☐	MRI-based neuroimaging

Animals and other organisms

Policy information about studies involving animals; ARRIVE guidelines recommended for reporting animal research

Laboratory animals	Experiments were conducted on larvae of the South African clawed toad Xenopus laevis of either sex, obtained from the Xenopus Biology Resources Centre (University of Rennes 1, France; http://xenopus.univ-rennes1.fr/). Animals were maintained at 20–22 °C in filtered aquarium water with a 12:12h light/dark cycle. Experiments were performed on tadpoles between developmental stage 40 and 58.
Wild animals	*Provide details on animals observed in or captured in the field; report species, sex and age where possible. Describe how animals were caught and transported and what happened to captive animals after the study (if killed, explain why and describe method; if released, say where and when) OR state that the study did not involve wild animals.*
Field-collected samples	*For laboratory work with field-collected samples, describe all relevant parameters such as housing, maintenance, temperature, photoperiod and end-of-experiment protocol OR state that the study did not involve samples collected from the field.*
Ethics oversight	All procedures were carried out in accordance with and were approved by the local ethics committee of University of Bordeaux (#2016011518042273 APAFIS #3612).

Note that full information on the approval of the study protocol must also be provided in the manuscript.
